# Robust and accurate detection and sizing of repeats within the *DMPK* gene using a novel TP-PCR test

**DOI:** 10.1038/s41598-019-44588-3

**Published:** 2019-06-04

**Authors:** Maike Leferink, Daphne P. W. Wong, Shiwei Cai, Minli Yeo, Jocelin Ho, Mulias Lian, Erik-Jan Kamsteeg, Samuel S. Chong, Lonneke Haer-Wigman, Ming Guan

**Affiliations:** 1 Department of Human Genetics, Radboud University Nijmegen Medical Center, Nijmegen, The Netherlands; 2 The BioFactory Pte Ltd, Singapore, Republic of Singapore; 3 Khoo Teck Puat – National University Children’s Medical Institute, National University Health System, Singapore, Republic of Singapore; 4 Department of Paediatrics, Yong Loo Lin School of Medicine, National University of Singapore, Singapore, Republic of Singapore; 5 Department of Laboratory Medicine, National University Hospital, Singapore, Republic of Singapore

**Keywords:** Neuromuscular disease, Genetic testing

## Abstract

Myotonic dystrophy type 1 is a multisystem disorder caused by the expansion of a trinucleotide repeat in the *DMPK* gene. In this study we evaluated the performance of the FastDM1^TM^ *DMPK* sizing kit in myotonic dystrophy type 1 testing. This commercially available triplet repeat-primed PCR based kit was validated using reference and clinical samples. Based on testing with 19 reference samples, the assay yielded repeat sizes within three repeats from the consensus reference length, demonstrating an accuracy of 100%. Additionally, the assay generated consistent repeat size information with a concentration range of template-DNA, and upon repetition and reproduction (CV 0.36% to 0.41%). Clinical performance was established with 235 archived prenatal and postnatal clinical samples, yielding results of 100% sensitivity (95% CI, 97.29% to 100%) and 100% specificity (95% CI, 96.19% to 100%) in classifying the samples into the respective genotype groups of 5–35 (normal), 36–50 (non-pathogenic pre-expansion), 51–150 (unstable intermediate-sized pathogenic) or >150 (unstable pathogenic) CTG repeats, respectively. Furthermore, the assay identified interrupted repeat expansions in all samples known to have interruptions, and also identified interruptions in a subset of the clinical samples.

## Introduction

Myotonic dystrophy type 1 (DM1; MIM#160900) is a multisystem disorder caused by abnormal expansion of a CTG repeat in the 3′ untranslated region of the dystrophia myotonica-protein kinase gene (*DMPK*; MIM*605377)^1–3^. The size of the abnormal expansion in affected individuals has a positive association with the severity of symptoms and an inverse correlation with age of disease onset^4^. The disorder is autosomal dominantly inherited and affects multiple systems including skeletal and smooth muscle, as well as eyes, heart, endocrine and central nervous system^5^. Upon transmission to offspring the repeat length may increase and lead to a more severe disease, a mechanism known as anticipation. The broad spectrum of clinical presentations of DM1 overlap extensively with those of other disorders; and molecular testing is essential for a definitive diagnosis^6^.

Existing EMQN (European Molecular Genetics Quality Network) best practice guidelines classify the *DMPK* CTG repeats into four size ranges based on repeat stability and disease phenotype^7^. (1) Range of 5 to 35 repeats (CTG_(5–35)_) as stable repeats present in healthy individuals with no DM1 phenotype (normal alleles). (2) Range of 36 to 50 repeats (CTG_(36–50)_) as repeats at risk of instability on transmission present in healthy individuals (non-pathogenic pre-expansion alleles). (3) Range of 51 to 150 repeats (CTG_(51–150)_) as unstable repeats present in individuals who are either asymptomatic or have minimal or classical DM1 (unstable intermediate-sized pathogenic alleles). (4) Range of more than 150 repeats (CTG_(>150)_) as unstable repeats associated with more severe forms of classical, juvenile or congenital DM1 (unstable pathogenic alleles). Consequently, the sizes of CTG repeats are critical parameters for clinical diagnosis, even though the boundaries in repeat lengths of the four groups should not be interpreted too rigidly due to the existence of a large inter-individual variability^7,8^.

Traditionally, the detection of CTG expansions included two tests i.e. standard PCR followed by fragment length analysis and Southern blot analysis^7^. The standard PCR technique, although sufficient in detecting the lower range of CTG expansions, is not reliable in amplifying repeats of over ~150 CTG repeats) and may fail to detect larger expansions when smaller alleles are preferentially amplified (allelic dropout)^6^. Therefore, samples in which only one repeat length is detected by PCR must be further analyzed for the possible presence of an expanded allele^7^. Southern blot analysis has been the gold standard for expanded allele detection, but is notoriously laborious, expensive and requires large amounts of DNA (in case of genomic DNA Southern blot) and has functional limitations in identifying interrupted alleles (in case of long-range PCR Southern blot). It is not surprising that this method has been replaced by alternative approaches such as triplet-repeat primed PCR (TP-PCR) in most diagnostic centers^7^.

Various TP-PCR based approaches have been developed and TP-PCR has proven to be an accurate technique in DM1 expansion analysis^7,9–11^. In the TP-PCR technology, (CAG)_n_ or (CTG)_n_ primers are used for the amplification of the repeat. These primers bind randomly to the repeat, and, in combination with a primer outside the repeat followed by PCR, will result in a pool of DNA fragments (Supplementary Fig. S1). Size separation of these fragments results in the specific ‘ladder’ or ‘comb-tooth’ pattern (Supplementary Fig. S1). This pattern is weaker in the longer repeat range and will quench due to technical issues, rather than that the end of the repeat expansion is reached. Consequently, many laboratory-derived assays fail to distinguish between the diagnostically important difference of the higher end intermediate pathogenic (CTG_(51–150)_) and large pathogenic (CTG_(>150)_) alleles, due to an extinction of the TP-PCR signal before 150 CTG’s are reached.

In 3–5% of the population the normally pure CTG repeat is interrupted by other repeat sequences, such as CCG or CGG repeats^12–14^. These repeat-interruptions may have effect on the melting and subsequent amplification of the template DNA (often the interruptions are CCG or CGG, increasing the CG-content). Additionally, the (CAG)_n_ or (CTG)_n_ repeat primers cannot bind to the interruption, thereby the quenching of the signal can be missed. Alleles with such interruption are thus prone to remain undetected or to be inaccurately sized^15^. The aim of the current study is to validate a commercial kit based on the bidirectional TP-PCR approach on its performance and utility in diagnostic DM1 testing.

## Results

### Sizing accuracy

The accuracy of the FastDM1^TM^ *DMPK* sizing kit was evaluated using 19 reference samples, covering the range of normal to unstable expanded alleles (Supplementary Table S1). For ten reference samples a consensus length was determined by three independent laboratories and the other nine were verified by one laboratory^16^. All repeat lengths determined by the *DMPK* sizing kit were within three repeats to the (consensus) repeat lengths of the study by Kalman *et al*.^16^. (Supplementary Table S1). In conclusion, a total agreement of 100% in sizing between the test results and the expected result was reached. It should be noted that a sizing cut-off of 180 repeats was used following the *DMPK* sizing kit manufacturer’s instructions. Consequently, alleles with repeat sizes equal to or beyond 180 repeats were all reported as having >180 repeats and considered to be in agreement with the expected results regardless of their exact sizes.

### Analytical sensitivity

The analytical sensitivity of the *DMPK* sizing kit was evaluated using four reference samples by examining the accuracy of the sizing results using varying amounts of DNA inputs of 1, 5, 10, 25, 50 and 200 ng. A reduction to 25 ng or increase to 200 ng of DNA input elicited no change on the detection or sizing accuracy for all alleles ranging from normal to unstable pathogenic repeats (Supplementary Table S2). A reduction to 10 ng or less DNA input elicited no change on the detection or sizing accuracy for all alleles except for the unstable intermediate-sized pathogenic allele in NA23378 in which the determined repeat length deviated with 5 or 6 repeats compared to the expected repeat length in either or both the 5′ and 3′ reactions.

### Detection of simulated mosaic samples

The analytic sensitivity and accuracy of the *DMPK* sizing kit in detecting mosaicism was evaluated using four series of simulated mosaic mixtures derived from eight reference samples with repeat sizes of 79, 475, ~1000 and ~1600 of the repeat expansion respectively. A mosaic level of 2.5% could be detected by both the 5′ and 3′ reactions, at 1% mosaicism the repeat expansion was missed by the 5′ reaction in case A (Supplementary Table S3). However, in the low mosaic range the sizing of the repeat expansion was not accurate. At a mosaic level of 10% or less, correct sizing of the repeat expansions was compromised by either the 5′ or 3′ reaction, or both. This indicates that mosaic samples can be reliably detected using the *DMPK* sizing kit, but that the repeat size can be underestimated at low (10% or less) levels of mosaicism.

### Analytical specificity

The analytical specificity of the *DMPK* sizing kit was in theory based on the distinctiveness of the primer sets designed for the test. Nevertheless, this aspect was additionally tested following a similar approach reported in a previous study^17^, using a DNA sample with a 58–65 CTG expansion in the unrelated *TCF4* gene. This *TCF4* gene CTG repeat did not influence the TP-PCR primers as two normal alleles of 10 and 13 CTG repeats in the *DMPK* gene were detected (Supplementary Fig. S2). Furthermore, the *TCF4* reference sample was added to each of four *DMPK* reference samples used in the sensitivity study and also in these samples the *DMPK* CTG repeat profiles were not altered by the *TCF4* expansion (Supplementary Table S4). These results demonstrated that the *DMPK* sizing kit can accurately size and genotype the *DMPK* alleles in the presence of other non-*DMPK* CTG repeats.

### Consistency

The study of consistency included intra-assay consistency, and intra- and inter-batch reproducibility. All consistency studies were conducted using four reference samples covering the respective genotypes of CTG_(5–35)_, CTG_(51–150)_ and CTG_(>150)_. For the intra-assay consistency, each sample was tested in ten replicates for each of the 5′ and the 3′ reactions, after which amplicons were subjected to two runs of capillary electrophoresis. Consequently, a total of 40 data points were obtained for each DNA sample tested. For intra-batch reproducibility, three kits: the first, middle and final kit; from a single production assembly process were sampled. Each kit was tested using both the 5′ and the 3′ reactions in at least twelve replicates. Finally, inter-batch reproducibility was determined using kits from three batches of production over a period of two years. The data set consisted of test results obtained by different operators using the same samples tested in duplicates or triplicates. The assay produced no variations in sizing for all alleles with repeat length less than 100. The variations observed were from the larger alleles of >100 repeats of sample NA23378 but with the respective coefficient of variations (CV) of no greater than 0.41% (Supplementary Table S5). Because the assay reports all alleles with triplet repeats more than 180 as >180 repeats, the CV calculation was not applicable for the large allele of NA04567 in the study.

### Performance on clinical samples

**Figure 1:**
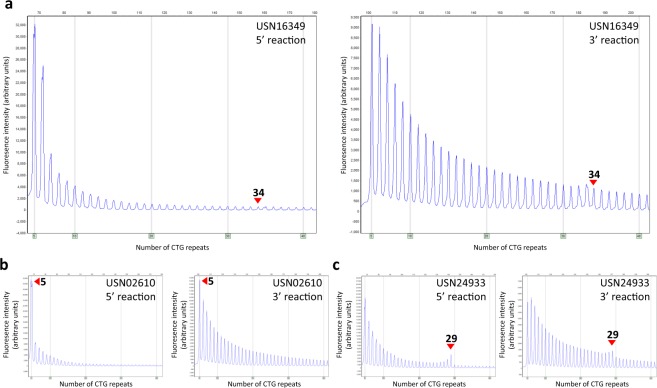
Electropherograms of the 5′ and 3′ *DMPK* sizing reactions of USN16349 (**a**), USN02610 (**b**) and USN24933 (**c**). In USN16349 the normal allele of 34 repeats was not visible and this sample was incorrectly interpreted to have a normal allele of 5 CTG repeats. The electropherogram of a random sample with a normal allele of 5 CTG repeats next to an expanded allele, USN02610, however, shows a different profile. The peak after the CTG repeat of 5 drops much stronger in USN02610 compared to USN16349. A normal allele of 29 next to an expanded allele, as in USN24933, is correctly identified.

The clinical performance of the *DMPK* sizing kit was examined using 225 postnatal and 10 prenatal clinical samples. The samples were previously characterised for *DMPK* CTG repeat length using a combination of methods including conventional PCR, TP-PCR and/or Southern blot of long-range PCR products. Firstly, the kit correctly sized 100% of the alleles in CTG_(5–35)_;CTG_(5–35)_ genotype samples (194/194 alleles in 94 postnatal and 3 prenatal cases) and 100% of the alleles in CTG_(5–35)_;CTG_(36–50)_ genotype samples (40/40 alleles in 20 postnatal cases) within 1 repeat of the reference results (Table 1). Secondly, 100% of the normal alleles and 44 unstable intermediate-sized pathogenic alleles in a total of 49 CTG_(5–35)_;CTG_(51–150)_ genotype samples (all postnatal) were sized within 5 repeats of the references (Table 1). The five unstable intermediate-sized pathogenic alleles that deviated by more than 5 repeats compared to the reference, USN31820, USN05133, USN16457, USN16779 and USN04608 (all postnatal samples), were originally sized as borderline alleles with repeats sizes approaching 150 repeats (more specifically 135, 140, 147, 149 and 140, respectively) while the *DMPK* sizing kit determined expansions of >180 repeats. To verify the correct repeat length in all five discrepant samples, they were additionally tested using Southern blot and were determined to have expanded alleles in the range of >180 repeats and relocated to CTG_(>150)_ for overall analysis (Table 2). Lastly, 68 normal alleles in a total of 69 CTG_(5–35)_;CTG_(>150)_ genotype samples (63 postnatal and 6 prenatal) were sized to within 1 repeat of the reference and all unstable alleles were determined to have a repeat length >150 (Table 1). One normal allele was misinterpreted to have 5 CTG repeats instead of 34. In this case (USN16349) the signal of the 34 repeat allele was masked by the expansion on the other allele (Fig. 1). In summary, the total agreement on sizing accuracy between the kit and the references were 99.7% (331/332 alleles) within 1 repeat for the normal alleles (<35 repeats) and 96.38% (133/138 alleles) within the same genotype group for the pre-expansion and unstable alleles (>35 repeats).

**Table 1:** Repeat size concordance of clinical samples using the *DMPK* sizing kit in comparison with the in-house methods at Radboud University Nijmegen Medical Center^7^.

Genotype	Sample size	Repeat Size Concordance			
		Allele 5–35rpts	Allele ≥36rpts		
		Within 1 repeat (±1)	Within 1 repeat (±1)	Within 5 repeats (±5)	Within same genotype group
CTG_(5–35)_; CTG_(5–35)_	97	194/194	—	—	—
CTG _(5–35)_; CTG _(36–50)_	20	20/20	20/20	20/20	20/20
CTG _(5–35)_; CTG _(51–150)_	49	49/49	31/49	44/49	44/49
CTG _(5–35)_; CTG _(>150)_	69	68/69*	N.A.^†^	N.A.^†^	69/69
Total Agreement		331/332 (99.70%)	51/69 (73.91%)	64/69 (92.75%)	133/138 (96.38%)

N.A. not applicable.

^*^In one CTG _(5–35)_; CTG _(51–150)_ genotype sample, the peak pattern of the 34 CTG normal allele was masked by the larger allele and was misinterpreted as carrying 5 CTG repeats.

^†^In all CTG _(5–35)_; CTG _(>150)_ genotype samples the exact length of the allele >150 rpts could not be determined in the reference test and *DMPK* sizing kit.

**Table 2:** Classification of clinical samples using the *DMPK* sizing kit in comparison with the in-house methods at Radboud University Nijmegen Medical Center^7^.

			Classification based on the reference methods at Radboud University Nijmegen Medical Center				
			Normal	Expanded			
			CTG _(5–35)_	CTG _(36–50)_	CTG _(51–150)_	CTG _(>150)_	
Classification using the commercial *DMPK* sizing kit	Normal	CTG _(**5–35**)_	97^TN^	0^FN^	0^FN^	0^FN^	Negative Predictive Value = 97/97 100%
	Expanded	CTG _(**36–50**)_	0^FP^	20^TP^	0^FN^	0^FN^	Positive Predictive Value = 138/138 100%
		CTG _(**51–150**)_	0^FP^	0^FP^	44^TP^	0^FN^	
		CTG _(**>150**)_	0^FP^	0^FP^	0^FP^	69 + 5^TP^*	
			Clinical Specificity = 97/97 100% (95% CI: 96.19–100%)	Clinical Sensitivity = 138/138 100% (95% CI: 97.29–100%)			

TN: True Negative; FN: False Negative; TP: True Positive; FP: False Positive.

*The five samples were originally sized under the CTG(51–150) genotype, but relocated into the CTG(>150) genotype upon additional Southern blot testing.

Nonetheless, the *DMPK* sizing kit correctly classified all the 235 clinical samples into their respective genotypes as [CTG_(5–35)_; CTG_(5–35)_] (n = 97), [CTG_(5–35)_/CTG_(36–50)_] (n = 20), [CTG_(5–35)_/CTG_(51–150)_] (n = 44) and [CTG_(5–35)_/CTG_(>150)_] (n = 74) (Table 2). Collectively, the overall findings indicate an overall performance of the kit in detecting the CTG expansions as having 100% clinical specificity and sensitivity (95% CI: 96.19% to 100% for specificity; 97.29% to 100% for sensitivity, respectively). The results also demonstrated the utility of the kit with 100% in positive predictive value (PPV) and negative predictive value (NPV) respectively (Table 2).

### Detection of CTG Repeats with interruptions in clinical samples

**Figure 2:**
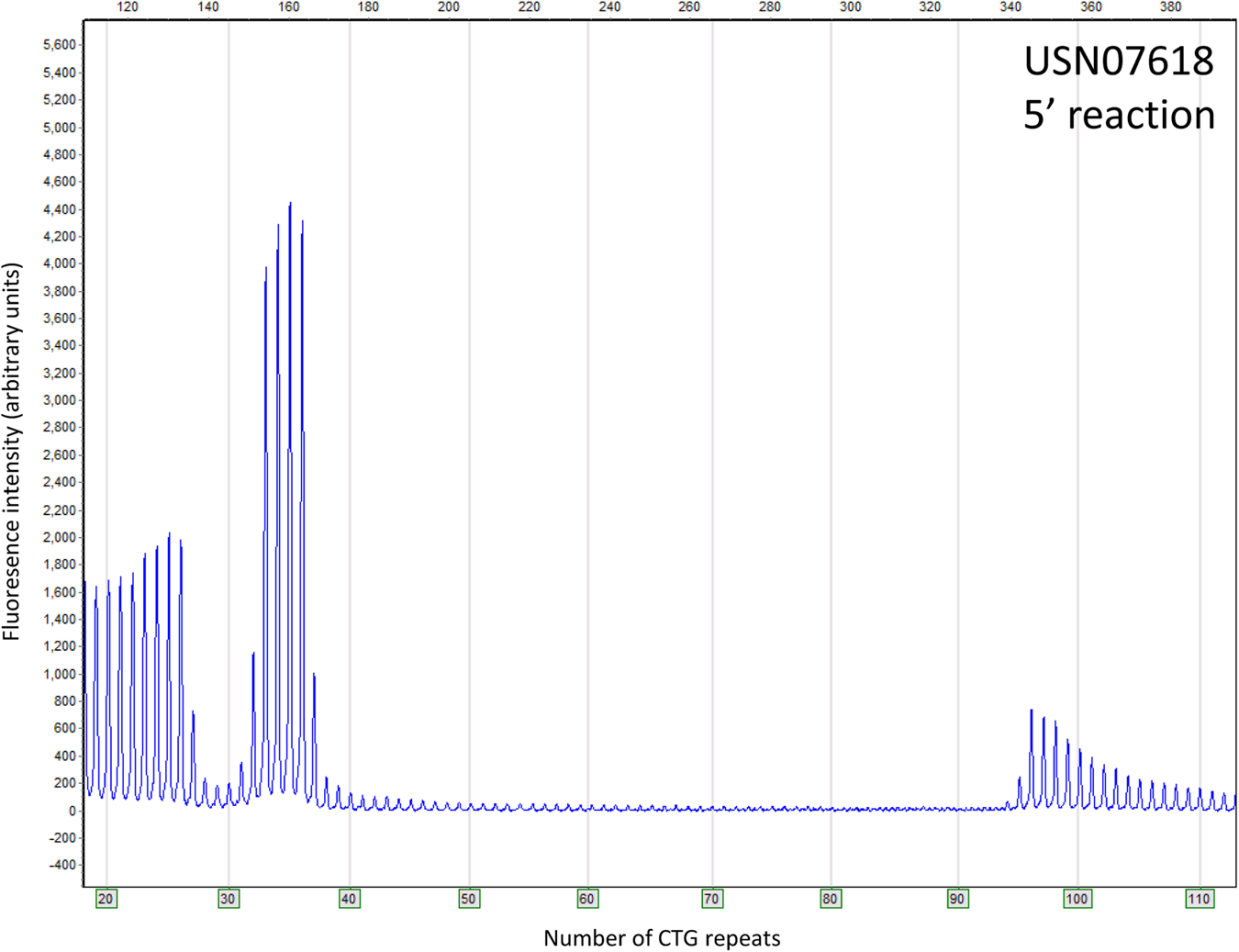
Electropherogram of the 5′ *DMPK* sizing reaction of USN07618. USN07618 was previously determined to have a repeat-interruption. The results of the *DMPK* sizing kit are indicative of the presence of an interruption, as gaps are present in the ladder/tooth-comb pattern since the (CAG)_n_ primer cannot anneal at the interruption.

Repeat-interruptions of the normally pure CTG repeat influence DNA melting, but more importantly, will not be a template for the (CAG)n or (CTG)n primers used in the TP-PCR techniques. To test whether repeats with interruptions are detected by the *DMPK* sizing kit, eight samples with previously observed interruptions were tested. In six samples the electropherogram patterns showed regions with gaps, indicative of the interruptions (Fig. 2). Although, USN03117 and USN01084 had continuous patterns in the electropherograms, the result of the *DMPK* sizing kit deviated from the non-interrupted repeat samples as in these samples the repeat length of the largest allele differed by 100 and 16 repeats between the 5′ and 3′ reactions, respectively (Table 3). Moreover, in the other six samples the repeat length determined by the 5′ and 3′ reactions also differed by more than 5 repeats (range 8–111; Table 3).

**Table 3:** Detection of non-CTG repeat interruption using the *DMPK* Sizing kit on eight samples with aberrant initial results.

Unique Study Number	Expected No. of repeats	Reaction	No. of CTG and non-CTG Repeats			
			Sizing Kit			Reference
			Allele 1	Allele 2*	5′-[CTGn-(XXXn)-CTGn]-3′^†^	Interruption pattern/Remarks
USN03117	12; >150	5′	12	229	ND	
		3′	12	199	ND	
USN04034	12; >150	5′	12	225	ND	
		3′	12	114	[39-(63)-12]	(CTG)_n_(CCGCTG)_~100_(CTG)_n_
USN08692	13; >150	5′	13	240	ND	
		3′	13	210	[147-(3)-6-(34)-20]	(CTG)_n_(CCGCTG)_114_(CTG)_n_
USN00144	5; 174	5′	5	154	[10-(2)-6-(79)-57]	
		3′	5	146	[10-(2)-6-(73)-54]	(CTG)_24_(CCGCTG)_45_(CTG)_60_
USN01084	13; >150	5′	13	94–105	ND	
		3′	13	78	ND	(CTG)_n_(CCGCTG)_167_(CTG)_n_
USN01299	13; >150	5′	13	259	[193-(50)-16]	
		3′	13	190	[118-(4)-6-(49)-13]	(CTG)_n_(CCGCTG)_37_(CTG)_n_
USN06424	5; >150	5′	5	253	ND	
		3′	5	161	[80-(43)-38]	
USN07618	14; >150	5′	14	205	[26-(2)-8-(55)-113]	
		3′	14	105–135	ND	

*The number of repeats was based on the presence of peaks (the approximation approach) and for large expanded alleles was the actual count until peaks diminished without applying the cut-off to facilitate the analysis of non-CTG repeats.

^†^ND: No detection of interruptions.

Additionally, in 16 of the 235 clinical samples repeat interruption’s were detected by the *DMPK* sizing kit. These were detected in seven samples with normal alleles (CTG_(5–35)_) and nine samples with an unstable pathogenic allele (CTG_(>150)_). In all seven interrupted CTG_(36–50)_ samples, interruptions were detected by both 5′ and 3′ reactions. Of note, based on sample (USN12581) that was additionally verified by Sanger sequencing to have (CTG)_6_(CCGCTG)_14_(CTG)_5_, the 5′ reaction of the kit correctly detected the exact interruption with the flanking CTG repeats (Supplementary Table S6). In contrast, in seven out of nine samples with an interrupted CTG_(>150)_ allele gaps were only present in the pattern of either the 5′ or 3′ reaction (Supplementary Table S6). In all interrupted CTG_(>150)_ alleles the repeat length of the largest allele determined by the 5′ and 3′ reactions deviated by more than 5 repeats, while there was no difference in repeat length in the interrupted CTG_(5–35)_ samples (Supplementary Table S6). In conclusion, the sizing kit is able to detect interrupted repeats in the *DMPK* gene, although sizing of alleles with interruptions may be less accurate.

## Discussion

The accuracy in sizing the CTG repeats is undeniably a critical parameter in DM1 testing even though the boundaries in repeat lengths should not be taken too rigidly predicting disease onset or severity^7^. For all 19 reference samples (39 alleles; due to ‘mosaicism’ in one patient having one normal allele (12 repeats) and two distinct population of repeat expansion of 56 and 70 repeats, Supplementary Table S1), detected repeat lengths where within 3 repeats to the (consensus) repeat length determined in the study by Kalman *et al*.^16^, reaching an accuracy of 100%. Next, the analytical sensitivity, analytical specificity and consistency of the *DMPK* sizing kit were assessed. Firstly, the length of the CTG repeat could be correctly identified using a DNA input of 25 to 200 ng. Furthermore, a mosaic level of up to 1% was identified by either the 5′ or 3′ reaction, whereas correct sizing of the repeat length was compromised below 20% mosaicism only. Secondly, a sample with 58–65 CTG repeats in the *TCF4* gene did not interfere with the *DMPK* sizing result. Thirdly, the consistency of the *DMPK* kit was established to have a coefficient of variation of <0.41% for intra-assay, intra-batch or inter-batch variations.

To complete the assessment of the performance of the *DMPK* sizing kit 235 clinical samples were tested. All normal alleles regardless of the genotype (in total 332 alleles) were detected within 1 repeat of the references with one exception. In this case (USN16349) the CTG repeat length was incorrectly sized using the *DMPK* sizing kit as 5 CTG repeats instead of 34. This was the largest normal allele tested in a sample that also has a CTG_(>150)_ allele. The second largest normal allele of 29 CTG repeats on one allele next to a pathogenic expansion on the other allele was correctly identified. In retrospect, the electropherogram of USN16349 is also not indicative for a repeat length of 5 (Fig. 1). It is anticipated that such aberrant patterns will be identified when the operators will become more familiar with the kit. More importantly, this sample was categorized into the correct genotype group of [CTG_(5–35)_; CTG_(>150)_], as the expanded allele was detected. Of the 140 alleles with a repeat length above 35, 5 differed by more than 5 repeats from the original determined length. Southern blot analyses of these 5 cases showed that the length of >180 determined by the *DMPK* sizing kit was most likely correct. The improvement of the *DMPK* sizing kit over the original tests in these samples is most likely due to the improved identification of longer repeats lengths (up to around 230–250 repeats), whereas the reference approach relied on a PCR that has a sizing limit of approximately 125–150 repeats^7^. Somatic heterogeneity of CTG repeat length is a common feature in blood cells of myotonic dystrophy patients^18^ and Southern blot analysis of these samples showed indeed somatic heterogeneity. The incorrect sizing by the historical test is most likely due to the preferential amplification of the shorter expanded alleles by the PCR of the reference method. Ultimately, the *DMPK* sizing kit classified all the clinical samples accurately into the respective allelic classes and yielded an overall performance of 100% in both sensitivity and specificity.

As the CTG repeat in *DMPK* can increase significantly upon transmission to off-spring thereby possibly causing a fatal congenital disease, prenatal testing is often requested for DM1^19^. The 235 clinical samples consisted of 10 prenatal samples derived from amniotic cells or chorionic villus with repeat length in the genotypes of CTG_(5–35)_ and CTG_(>150)_. In all prenatal samples the CTG repeat length was correctly determined.

Interruptions of the normally pure CTG repeat in the *DMPK* gene have been described in 3–5% of expanded alleles^12–14^. Due to the nature of the interruptions, CTG/CAG primers in the TP-PCR will not anneal to the interrupted regions and higher G/C content may also compromise PCR and result in allelic dropouts. In six out of eight samples with previously identified interruptions, repeat expansions with interruptions were easily detected using the *DMPK* sizing kit, due to gaps in the electropherogram patterns. In the additional two samples there was a clear indication of the presence of an interruption due to the difference of more than 5 repeats between the 5′ and 3′ reaction. Unlike the pathogenic expansions without interruptions, differences between the 5′ and 3′ reactions were well above 5 repeats in the interrupted pathogenic expansions. The large differences in sizes are likely due to the non-CTG repeat tracts impairing the identification of the complete repeat in either or both the 5′ and/or 3′ reactions. An interrupted expansion may be sized inaccurately by TP-PCR if the interruption extends beyond the detection limit of the TP-PCR at both ends of the expanded tract^15^. Case USN01084 fits the above description, as a continuous electropherogram pattern was present in both the 5′ and 3′ reactions, suggestive of a CTG repeat size of 94–105 or 78, respectively. Nevertheless, the difference in repeat length between the 5′ and the 3′ reactions was noticeably larger than what could be attributed solely to variations of assays in design, suggestive of an interruption located in the approximate middle of the repeat. Thus, when the CTG repeat length determined by the 5′ and 3′ reaction deviates by more than five repeats, most likely an interrupted repeat is present and additional tests (such as Southern blotting) should be performed to characterize the repeat length. However, if the repeat length is determine to be >150 by one or both the 5′ and 3′ reactions, the repeat should be assigned as an unstable pathogenic allele and further characterization will have no clinical consequence.

Four of the interrupted CTG_(>150)_ repeats with aberrant electropherogram patterns were previously characterized by enzyme digestion and Southern blot. These results deviated from the results determined by the *DMPK* sizing kit in number and location of the interruptions. One obvious reason for the difference is that the reference approach was more specific with types of sequences recognized by the restriction enzyme, whereas primer binding in TP-PCR is affected by any repeat interruption. Long-read sequencing may determine the exact sequence of these interrupted alleles in the future. Initial studies on limited numbers of patients have suggested that interrupted repeat expansions are more stable and DM1 patients with interrupted repeat expansions have a later disease onset compared to DM1 patient with pure CTG repeat expansions of the same length^13,14,20,21^. However, the poor individual correlation between repeat length and disease severity and the large heterogeneity in type, location and length of repeat interruptions makes studies on large groups of DM1 patients with interrupted repeats necessary to determine whether it is of prognostic value to determine if an expansion is interrupted and/or quantify the CTG and non-CTG repeat tracts of the interrupted expansions.

In conclusion, the FastDM1^TM^ *DMPK* sizing kit was found to be robust and accurate. The analytical sensitivity was not altered when the DNA input was varied to as low as a quarter or as high as twice the recommended amount. Similarly, the analytic specificity maintained well without generating any non-specific profiles even when DNA containing a non-*DMPK* CTG repeat expansion was introduced into the testing. The consistency of the kit was established as <0.41% CV for intra-assay, intra-batch and inter-batch variations. These parameters suggested that the kit performances will easily be obtainable at the user’s ends. The kit addressed the specific testing need for accurately categorizing samples into the respective genotypes (i.e. to clearly distinguish between repeats under and over 150 repeats). Moreover in 5 samples the kit correctly identified the unstable pathogenic allele (CTG_(>150)_), while a previously performed TP-PCR test incorrectly determined the presence of an unstable intermediate allele (CTG_(50–150)_). Hence the FastDM1^TM^ *DMPK* sizing kit represents an attractive alternative to current approaches in DM1 testing.

## Methods

### Reference DNA samples

Twenty-three reference samples of cell line-derived DNA from Coriell Cell Repositories (Camden, USA) were included in this study. Sizing accuracy was examined using 19 well characterized samples with sizing information and/or common consensus reported elsewhere^16^ (Supplementary Table S1). To examine the analytic sensitivity and specificity of the kit, four reference samples covering CTG repeats of different lengths from normal to large expanded alleles (NA03928, NA06075, NA23378 and NA04567) were selected. The analytic sensitivity of the kit was established using the four samples in varying amounts of DNA inputs at 1, 5, 10, 25, 50, 100 and 200 ng per reaction. The analytic specificity was evaluated using the same four samples (NA03928, NA06075, NA23378 and NA04567) with the introduction of another reference sample (NA20232) with a CTG repeat expansion in another gene. To evaluate the sensitivity of the kit in detecting low level mosaicism of expanded alleles, four series of mixtures were created using eight samples (NA16243/NA23258, NA03928/NA05152, NA23265/NA04033 and NA23378/NA23299) to simulate mosaic content. These samples were mixed in various proportions, yet maintained a total DNA input of 100 ng per reaction. The resulting simulated mosaic samples contained 1%, 2.5%, 5%, 10%, 20%, 50% and 100% of the allele with the longest repeat length. All samples were additionally checked for their quantity and purity using common practices. DNA concentration was estimated by absorbance of 260 nm whereas the purity evaluated by calculating the ratio of A260/A280.

### DNA Samples from clinical archive

The clinical performance of the sizing kit was validated using 235 archived clinical samples from the Radboud University Medical Center, which were anonymized and coded with an unique study number (USN). These consisted of 225 postnatal samples, source material whole blood (n = 224) or cord blood (n = 1) and 10 prenatal samples, source material amniotic cells (n = 5) or chorionic villus (n = 5). Furthermore, eight additional samples were tested with initial testing results indicative for the presence of interrupted repeat expansions. DNA was isolated using the fully automated Chemagic Star according to manufacturer’s recommendations (Perkin Elmer, Waltham, USA). The samples were characterised at Radboud University Nijmegen Medical Center for *DMPK* CTG repeat length using a combination of methods including conventional PCR, TP-PCR and/or Southern blot of long-range PCR products^7^. Five samples were additionally tested using Southern blot of long-range PCR products, digested with restriction enzyme (*Aci* I and *MspA* 1I; Prof D. Monckton unpublished data). Usage of archived DNA samples for validation purpose was permitted under the guidelines of the Radboud University Nijmegen Medical Center, Nijmegen, Netherlands.

### FastDM1^TM^ DMPK sizing kit

The FastDM1^TM^ *DMPK* sizing kits (labelled “For Research Use Only” (RUO)) were obtained from The BioFactory Pte Ltd (Singapore, Singapore). The kit was developed based on previous studies by Lian *et al*.^10,22^. It utilizes a triplet-primed polymerase chain reaction, which includes a combination of primers targeting a flanking region as well as five CTG repeats. A schematic figure of the reaction is shown in Supplementary Fig. S1. Primer mixes were designed to amplify from the 5′ and 3′ ends of the *DMPK* CTG repeat region.

The TP-PCR assays of the kit were performed according to manufacturer’s instructions in 15 μL volumes with 100 ng of genomic DNA per test, unless otherwise stated. The 5′ and 3′ TP-PCR reactions were carried out in separate tubes using a Bio-Rad C1000 (Bio-Rad, Hercules, USA) or Veriti (Applied Biosystems, Foster City, USA) thermal cycler. The TP-PCR comprised of an initial denaturation step at 95 °C for 15 min followed by 35 cycles of 98 °C for 45 sec, 60 °C for 45 sec and 72 °C for 5 min, with a final extension step at 72 °C for 10 min.

TP-PCR amplification products were analysed by capillary electrophoresis (CE). Briefly, a 2 µL aliquot of each amplicon was mixed with 0.5 µL of MapMarker^®^1000-ROX (Bioventures Inc, Murfreesboro, USA) or GeneScan™ 1200 LIZ^™^ (Applied Biosystems) size standard and 9 µL of Hi-Di™ formamide (Applied Biosystems). The mixture was denatured at 95 °C for 5 min. The mixture was analyzed on an ABI 3730xL DNA Analyzer (Applied Biosystems) with a capillary length of 50 cm and running POP-7 polymer. CE settings were an injection time of 5 sec at 3 kV or 1 kV for LIZ^™^ and capillary electrophoresis for 50 min at 15 kV.

### Data analysis and result interpretation

Electropherograms were analysed with Peakscanner software (version 2.0, Applied Biosystems) or Genemarker software (version 2.6.7, SoftGenetics, State College, USA), following manufacturer’s instructions. Briefly, the electropherograms were visually checked for aberrant results (e.g. contamination in the no template controls, or incorrect calling of size standard peaks) and valid electropherograms were subjected to repeat sizing analysis. The final peak of each sample was identified based on signal height and/or peak morphology. The repeat size was determined by counting the first until the respective final peak(s) or by approximation based on correction factor calculated using distance between the two peaks in base pairs. In this way, the amount of CTG repeats was determined ranging from 5 till 180, and repeats longer than 180 were classified as >180. Samples for the clinical performance testing were classified into the respective genotypes (i.e. CTG_(5–35)_, CTG_(36–50)_, CTG_(51–150)_ and CTG_(>150)_) based on the reported repeat size of the largest allele, following the EMQN guidelines^7^. Of note, 95% confidence intervals of specificity and sensitivity were calculated using Wilson score interval. Coefficient of variation (CV) was used for the study of consistency and was measured by the ratio of the standard deviations (SD) to the mean (SDs over the averages of the respective sets of data points). Furthermore, aberrant patterns or significant disagreements between the 5′ and 3′ TP-PCR reaction samples were interpreted as indicative of “non-CTG repeat” interruptions or rare deletion on one end of the CTG repeat region. For the study of “non-CTG repeat” interruptions, repeats of large expanded alleles were counted until peak signal diminished without applying the cut-off (i.e. carry on counting beyond 180 repeats). The size of “non-CTG repeats” was calculated based on the absence of peaks using the difference in base pairs between the adjacent peaks. Example electropherograms of pure CTG and interrupted CTG repeats of the 5′ and 3′ DMPK sizing reaction of clinical samples with different repeat lengths are shown in Supplementary Fig. S3.

Data analysis was performed blinded by a technician and clinical laboratory geneticist in training. Their results were compared to each other and when the results did not match the sample was re-examined until a joint agreement was reached. Only then were results compared to the results of the original test.

## Supplementary information

Supplementary information

## Data Availability

The datasets generated and/or analysed during the current study are available from the corresponding author on reasonable request.
