# Supplementary material for: Robust and accurate detection and sizing of repeats within the DMPK gene using a novel TP-PCR test
Source: Sci Rep. 2019 Jun 4;9:8280. doi: 10.1038/s41598-019-44588-3 (PMC6547747; doi:10.1038/s41598-019-44588-3)
Supplement: Supplementary file 1 — Supplementary information [file 41598_2019_44588_MOESM1_ESM.docx]

*Supplementary information:*

**Robust and accurate detection and sizing of repeats within the *DMPK* gene using a novel TP-PCR test**

Maike Leferink^1&^, Daphne P. W. Wong^2&^, Shiwei Cai^2^, Minli Yeo^2^, Jocelin Ho^2^, Mulias Lian^3^, Erik-Jan Kamsteeg^1^, Samuel S. Chong^3-5^, Lonneke Haer-Wigman^1*#^, and Ming Guan^2#^

^1^Department of Human Genetics, Radboud University Nijmegen Medical Center, Nijmegen, The Netherlands;

^2^The BioFactory Pte Ltd, Singapore;

^3^Khoo Teck Puat – National University Children’s Medical Institute, National University Health System, Singapore;

^4^Department of Paediatrics, Yong Loo Lin School of Medicine, National University of Singapore, Singapore;

^5^Department of Laboratory Medicine, National University Hospital, Singapore

^&^Both junior authors contributed equally to this work.

^#^Both senior authors contributed equally to this work

*Corresponding author: Lonneke Haer-Wigman, Lonneke.Haer-Wigman@radboudumc.nl.

**Supplementary Figure S1**

Schematic overview of the principle of the FastDM1^TM^ *DMPK* sizing 5’and 3’ reactions. F: *DMPK* specific forward primer, R: *DMPK* specific reverse primers. The blue star depicts the fluorescent label of the *DMPK* specific primers. In the TP-PCR technology, (CAG)_n_ or (CTG)_n_ primers are used for the amplification of the repeat. These primers bind randomly to the repeat, and, in combination with the *DMPK* specific primer outside the repeat followed by PCR, will result in a pool of DNA fragments. Size separation of these fragments results in the specific ‘ladder’ or ‘comb-tooth’ pattern. Normal, non-pathogenic pre-expansion and unstable intermediate-sized pathogenic alleles will have an increase in fluorescent signal of the repeat length that is present in the patient. This pattern is weaker in the unstable pathogenic repeat range and will quench due to technical issues, rather than that the end of the repeat expansion is reached, not shown in this schematic overview.

**Supplementary Figure S2**

Electropherograms of NA20232 on a CTG-repeat assay specific for *TCF4* (**a**) and *DMPK* 5’ reaction (**b**) and 3’ reactions (**c**). **a**) Alleles of 18 and 58-56 were detected in the *TCF4* gene for NA20232. **b** and **c**) Alleles of 10 and 13 repeats in the *DMPK* gene were detected in NA20323, no elongation of the signal to 18 or to 58-56 was observed.

**Supplementary Figure S3**

Electropherogram of the 5’ and 3’ *DMPK* sizing reactions of USN14101 (**a** and **b**), USN03583 (**c**), USN18379 (**d**), USN23467 (**e**), USN19090 (**f**), USN32388 (**g**) and a negative control (**h**). USN14101 has a homozygous *DMPK* CTG repeat length of 13 (**a**), and only background noise is observed in the size range between 100 and 200 repeats (**b**). USN03583 has a *DMPK* CTG repeat length of 13 and 46 (**c**). USN18379 has an interrupted unstable non-pathogenic *DMPK* CTG repeat (**d**). USN23467 has a *DMPK* CTG repeat length of 5 and 147 (**e**). USN19090 has a *DMPK* CTG repeat length of 14 and >180 (**f**). USN32388 has an interrupted *DMPK* CTG repeat length just over 180 (**g**). In the negative control only background noise is detected, the fluorescence intensity is far lower compared to the fluorescence intensity from DNA samples (**h**).

**Supplementary Table S1** CTG repeat sizes of reference samples and determined in the accuracy study of the *DMPK* Sizing kit.

|  | **No. of CTG repeats** | | | | | | |
| --- | --- | --- | --- | --- | --- | --- | --- |
|  | **Expected (X)** | | |  | **Difference (Y-X)** ^‡^ | | |
|  |  | **Kalman et al^16^**^†^ | |  |  | **Kalman et al^16^** | |
| **Coriell Sample ID** | **Coriell*** | **Lab 1** | **Consensus** | **This study (Y)** ^#^ | **Coriell** | **Lab 1** | **Consensus** |
| NA06075 | N; 66 | 12; 55; 69 | 12; 56; 70 (±0.9) | 12; 56; 71 | 0^N^, +5 | 0, +1, +2 | 0, 0, +0.1 |
| NA23265 | 12; 60 | 12; 75 | 12; 75 | 12; 77 | 0, +17 | 0, +2 | 0, +2 |
| NA23378 | N; ~ 80-90 | 22; 145 | 22; 138 (±5) | 22; 132 | 0^N^, +42 | 0, -13 | 0, -1 |
| NA05164 | 21; ~ 340 | 21; 325 | 21; 377 (±53) | 21; >180 | 0, 0^E^ | 0, 0^E^ | 0, 0^E^ |
| NA03697 | ?; ~ 500 | 12; 370 | 12; 412 (±33) | 12; >180 | 0^N^, 0^E^ | 0, 0^E^ | 0, 0^E^ |
| NA04567 | N; ~ 700 | 21; 630 | 21; 637 (±33) | 21; >180 | 0^N^, 0^E^ | 0, 0^E^ | 0, 0^E^ |
| NA03696 | N; ~ 1000 | 12; 700 | 12; 697 (±13) | 12; >180 | 0^N^, 0^E^ | 0, 0^E^ | 0, 0^E^ |
| NA04648 | N; ~ 1000 | 5; 950 | 5; 1008 (±49) | 5; >180 | 0^N^, 0^E^ | 0, 0^E^ | 0, 0^E^ |
| NA05152 | N; ~ 1500 | 5; 1600 | 5; 1621 (±30) | 5; >180 | 0^N^, 0^E^ | 0, 0^E^ | 0, 0^E^ |
| NA03132 | 5; ~ 1700 | 5; 1950 | 5; 2078 (±217) | 5; >180 | 0, 0^E^ | 0, 0^E^ | 0, 0^E^ |
| NA03990 | ?; 50-80 | ?; 78 |  | 14; 79 | 0^N^, -1 | 0^N^, +1 |  |
| NA23258 | ?;70-80 | ?; 79 |  | 13; 82 |  | 0^N^, +3 |  |
| NA03756 | ?; ~ 500 | ?; 450 |  | 13; >180 | 0^N^, 0^E^ | 0^N^, 0^E^ |  |
| NA23374 | N; ~130-140 | ?; 475 |  | 5; >180 | 0^N^, + >40 | 0^N^, 0^E^ |  |
| NA23299 | N; 90-100 | ?; 475 |  | 22; >180 | 0^N^, + >80 | 0^N^, 0^E^ |  |
| NA23300 | N; 150-160 | ?; 550 |  | 5; >180 | 0^N^, + >20 | 0^N^, 0^E^ |  |
| NA03986 | ?; ~500 | ?; 550 |  | 12; >180 | 0^N^, 0^E^ | 0^N^, 0^E^ |  |
| NA04034 | ?; ~1000 | ?; 700 |  | 12; >180 | 0^N^, 0^E^ | 0^N^, 0^E^ |  |
| NA23256 | ?; 160-170^‡^ | ?; 775 |  | 25; >180 | 0^N^, + >10 | 0^N^, 0^E^ |  |

* CTG repeat sizes from Coriell; N, normal; ?, no data available

^†^ Based on data from Kalman et al**^16^** with consensus or without (by Lab 1)

^‡^  0^N^, differences in repeat sizes cannot be calculated due to the lack of sizing data from Coriell for the alleles; 0^E^, differences for large expanded alleles are calculated only up to the limit of 180 repeats, all expanded alleles with repeats exceeding 180 repeats by the references are considered concordant when the test reports as “>180 repeats”.

^#^ Sizes reported here were obtained followed the manufacturer’s instruction based the sizes identified by either 3’ or 5’ TP-PCR whichever were longer. In all except for 2 cases (NA23265, NA23378), the results by the 3’ or 5’ TP-PCR were identical. NA23265 was determined as 76 and 77; whereas NA23378 as 129 and 132 repeats respectively by the 3’ and 5’ TP-PCR.

**Supplementary Table S2** Repeat lengths detected at various amounts of input DNA.

| **Coriell Sample ID** | **Genotype** | **No. of CTG Repeats** | | | | | | | | | | | | | | | | |
| --- | --- | --- | --- | --- | --- | --- | --- | --- | --- | --- | --- | --- | --- | --- | --- | --- | --- | --- |
|  |  |  | **Expected*** **(X)** | ***DMPK* Sizing Kit (Y)** | | | | | | |  | **Difference (Y-X)**^†^ | | | | | | |
|  |  |  |  | **200 ng** | **100 ng** | **50 ng** | **25 ng** | **10 ng** | **5 ng** | **1 ng** |  | **200ng** | **100ng** | **50ng** | **25ng** | **10ng** | **5 ng** | **1 ng** |
| NA03928 | CTG_(5-35)_ | Allele 1 | 5 | 5 | 5 | 5 | 5 | 5 | 5 | 5 |  | 0 | 0 | 0 | 0 | 0 | 0 | 0 |
|  |  | Allele 2 | 12 | 12 | 12 | 12 | 12 | 12 | 12 | 12 |  | 0 | 0 | 0 | 0 | 0 | 0 | 0 |
| NA06075 | CTG _(51-150)_ | Allele 1 | 12 | 12 | 12 | 12 | 12 | 12 | 12 | 12 |  | 0 | 0 | 0 | 0 | 0 | 0 | 0 |
|  |  | Allele 2 | 56 | 56 | 56 | 56 | 56 | 56 | 56 | 56 |  | 0 | 0 | 0 | 0 | 0 | 0 | 0 |
|  |  | Allele 3 | 71 | 71 | 71 | 71 | 71 | 71 | 71 | 71 |  | 0 | 0 | 0 | 0 | 0 | 0 | 0 |
| NA23378 | CTG _(51-150)_ | Allele 1 | 22 | 22 | 22 | 22 | 22 | 22 | 22 | 22 |  | 0 | 0 | 0 | 0 | 0 | 0 | 0 |
|  |  | Allele 2 | 129 (5’) | 129 (5’) | 129 (5’) | 129 (5’) | 129 (5’) | 134(5’) | 135 (5’) | 134 (5’) |  | 0 | 0 | 0 | 0 | 5 | 6 | 5 |
|  |  |  | 132 (3’) | 132 (3’) | 132 (3’) | 132 (3’) | 132 (3’) | 132 (3’) | 132 (3’) | 134 (3’) |  | 0 | 0 | 0 | 0 | 0 | 0 | 2 |
| NA04567 | CTG _(>150)_ | Allele 1 | 21 | 21 | 21 | 21 | 21 | 21 | 21 | 21 |  | 0 | 0 | 0 | 0 | 0 | 0 | 0 |
|  |  | Allele 2 | >180 | >180 | >180 | >180 | >180 | >180 | >180 | >180 |  | N/A | N/A | N/A | N/A | N/A | N/A | N/A |

* Expected based on result obtained using optimal assay conditions, following manufacturer's instructions.

^†^ N/A: Not applicable

**Supplementary Table S3** Mosaicism (simulated) detection of the *DMPK* sizing kit.

| **Simulated Mosaic Sample** | | **CTG repeats** | **No. of CTG Repeats for the Largest Expanded Allele in the mixture** | | | | | | | | | | | | | | | | |
| --- | --- | --- | --- | --- | --- | --- | --- | --- | --- | --- | --- | --- | --- | --- | --- | --- | --- | --- | --- |
|  |  |  | **Expected**^*^ **(X)** | **Assay** | **FastDM1™ *DMPK* Sizing Kit (Y)** | | | | | | |  | **Difference (Y-X)** ^†^ | | | | | | |
|  |  |  |  |  | **100%** | **50%** | **20%** | **10%** | **5%** | **2.5%** | **1%** |  | **100%** | **50%** | **20%** | **10%** | **5%** | **2.5%** | **1%** |
| A | NA16243 | 13; 14 | 82 | 5' | 82 | 81 | 82 | 82 | 82 | 82 | ND |  | 0 | -1 | 0 | 0 | 0 | 0 | ND |
|  | NA23258 | 13; 82 |  | 3' | 82 | 82 | 83 | 82 | 82 | 82 | 83 |  | 0 | 0 | 1 | 0 | 0 | 0 | 1 |
| B | NA03928 | 5; 12 | >180 | 5' | >180 | >180 | >180 | >180 | >180 | 169 | 139 |  | N/A | N/A | N/A | N/A | N/A | -11 | -41 |
|  | NA05152 | 5; ~1621 |  | 3' | >180 | >180 | >180 | >180 | >180 | >180 | 161 |  | N/A | N/A | N/A | N/A | N/A | N/A | -19 |
| C | NA23378 | 22; ~138 | >180 | 5' | >180 | >180 | >180 | >180 | 174 | 162 | 164 |  | N/A | N/A | N/A | N/A | -6 | -18 | -16 |
|  | NA23299 | 22; ~475 |  | 3' | >180 | >180 | >180 | >180 | >180 | 171 | 169 |  | N/A | N/A | N/A | N/A | N/A | -9 | -11 |
| D | NA23265 | 12; 76 | >180 | 5' | >180 | >180 | >180 | >180 | >180 | 149 | 125 |  | N/A | N/A | N/A | N/A | N/A | -31 | -55 |
|  | NA04033 | 12; ~1000 |  | 3' | >180 | >180 | >180 | 172 | 158 | 139 | 131 |  | N/A | N/A | N/A | N/A | N/A | -41 | -49 |

* Expected based on results obtained using optimal assay conditions, following manufacturer's instructions.

^†^ N/A: Not applicable; ND: No detection of the low abundance allele.

**Supplementary Table S4** Analytical Specificity of the *DMPK* sizing kit, by addition of a non-relevant sample (NA20232).

| **Coriell Sample ID** |  | **No. of CTG Repeats** | | | | | | | |
| --- | --- | --- | --- | --- | --- | --- | --- | --- | --- |
|  |  | **Expected (X)** | ***DMPK* sizing Kit (Y)** | | |  | **Difference (Y-X)*** | | |
|  |  |  | **0ng** | **100ng** | **200ng** |  | **0ng** | **100ng** | **200ng** |
| NA03928 | Allele 1 | 5 | 5 | 5 | 5 |  | 0 | 0 | 0 |
|  | Allele 2 | 12 | 12 | 12 | 13^†^ |  | 0 | 0 | 0 |
| NA06075 | Allele 1 | 12 | 12 | 13^†^ | 13^†^ |  | 0 | 0 | 0 |
|  | Allele 2 | 56 | 56 | 56 | 56 |  | 0 | 0 | 0 |
|  | Allele 3 | 71 | 71 | 71 | 71 |  | 0 | 0 | 0 |
| NA23378 | Allele 1 | 22 | 22 | 22 | 22 |  | 0 | 0 | 0 |
|  | Allele 2 | 129 (5’) | 129 (5’) | 129 (5’) | 130 (5’) |  | 0 | 0 | +1 |
|  |  | 132 (3’) | 132 (3’) | 132 (3’) | 131 (3’) |  | 0 | 0 | -1 |
| NA04567 | Allele 1 | 21 | 21 | 21 | 21 |  | 0 | 0 | 0 |
|  | Allele 2 | >180 | >180 | >180 | >180 |  | N/A | N/A | N/A |

* N/A: Not applicable.

^†^  The expected 12 repeats allele were masked by the presence of the closely sized allele of 13 repeats from NA20232.

**Supplementary Table S5** Precision of the *DMPK* sizing kit, using genomic reference samples.

|  | |  | **No. of CTG Repeats** | | | | | | | | | | |
| --- | --- | --- | --- | --- | --- | --- | --- | --- | --- | --- | --- | --- | --- |
| **Coriell Sample ID** | **Expected** | | **Intra- Assay Variation (n=40)*** | | |  | **Inter-Assay Variation**^†^ | | | | | | |
|  |  |  |  |  |  |  | **Intra- batch Repeatability**  **(n=12 or 18)*** | | |  | **Inter- batch Reproducibility**  **(n=36 or 54)*** | | |
|  |  |  | **Avg.** | **SD** | **CV (%)** |  | **Avg.** | **SD** | **CV (%)** |  | **Avg.** | **SD** | **CV (%)** |
| NA03928 | Allele 1 | 5 | 5 | 0 | 0 |  | 5 | 0 | 0 |  | 5 | 0 | 0 |
|  | Allele 2 | 12 | 12 | 0 | 0 |  | 12 | 0 | 0 |  | 12 | 0 | 0 |
| NA06075 | Allele 1 | 12 | 12 | 0 | 0 |  | 12 | 0 | 0 |  | 12 | 0 | 0 |
|  | Allele 2 | 56 | 56 | 0 | 0 |  | 56 | 0 | 0 |  | 56 | 0 | 0 |
|  | Allele 3 | 71 | 71 | 0 | 0 |  | 71 | 0 | 0 |  | 71 | 0 | 0 |
| NA23378 | Allele 1 | 22 | 22 | 0 | 0 |  | 22 | 0 | 0 |  | 22 | 0 | 0 |
|  | Allele 2 | 129 (5’) | 129 | 0.47 | 0.36 |  | 129 | 0.53 | 0.41 |  | 129 | 0.49 | 0.38 |
|  |  | 132 (3’) | 132 | 0.47 | 0.36 |  | 132 | 0.53 | 0.4 |  | 132 | 0.51 | 0.39 |
| NA04567 | Allele 1 | 21 | 21 | 0 | 0 |  | 21 | 0 | 0 |  | 21 | 0 | 0 |
|  | Allele 2 | ≥180 | ≥180 | N/A | N/A |  | ≥180 | N/A | N/A |  | ≥180 | N/A | N/A |

* N/A: Not applicable.

^†^  The numbers of data points here: 12 and 36 are applicable to NA03928 and NA04567, whereas 18 and 54 are applicable to NA06075 and NA23378. For allele 2 of NA23378, the results are analysed separately in the 3’ and 5’ assay. Hence, the numbers of data points are half as what are listed.

**Supplementary Table S6** Non-CTG repeat interruptions detected in the study of 235 clinical archived samples.

| **Unique Study Number** | **Expected No. of CTG repeats** | **Assay** | **No. of CTG and non-CTG Repeats** | | |
| --- | --- | --- | --- | --- | --- |
|  |  |  | **Allele 1** | **Allele 2*** | **5'-[CTGn-(XXXn)-CTGn]-3'**^†^ |
| USN02204 | 12; 36 | 5’ | 12 | 37 | [6-(26)-5] |
|  |  | 3’ | 12 | 35 | [6-(24)-5] |
| USN02206 | 12; 36 | 5’ | 12 | 37 | [6-(26)-5] |
|  |  | 3’ | 12 | 35 | [6-(24)-5] |
| USN18377 | 5; 37 | 5’ | 5 | 38 | [6-(26)-5] |
|  |  | 3’ | 5 | 35 | [6-(24)-5] |
| USN18379 | 11; 37 | 5’ | 11 | 37 | [6-(26)-5] |
|  |  | 3’ | 11 | 35 | [6-(24)-5] |
| USN12581^‡^ | 14; 38 | 5’ | 14 | 39 | [6-(28)-5] |
|  |  | 3’ | 14 | 37 | [6-(26)-5] |
| USN09019 | 13; 42 | 5’ | 13 | 43 | [6-(32)-5] |
|  |  | 3’ | 13 | 40 | [6-(29)-5] |
| USN09410 | 13; 42 | 5’ | 13 | 43 | [6-(32)-5] |
|  |  | 3’ | 13 | 40 | [6-(29)-5] |
| USN01622 | 13; >150 | 5’ | 13 | 241 | ND |
|  |  | 3’ | 13 | 141 | [77-(42)-23] |
| USN05626 | 12; >150 | 5’ | 12 | 230 | [12-(1)-216] |
|  |  | 3’ | 12 | 183 | ND |
| USN14762 | 14; >150 | 5’ | 14 | 248 | ND |
|  |  | 3’ | 14 | 145 | [103-(1)-7-(1)-8-(1)-8-(1)-8-(1)-7] |
| USN15009 | 5; >150 | 5’ | 5 | 184-231 | ND |
|  |  | 3’ | 5 | 194 | [151-(1)-7-(1)-8-(1)-8-(1)-8-(1)-7] |
| USN19330 | 12;>150 | 5’ | 12 | 380 | 190 (75-(31)-8-(1)-75) |
|  |  | 3’ | 12 | 390 | 195 (78-(32)-8-(1)-76) |
| USN21764 | 13; >150 | 5’ | 13 | 241 | ND |
|  |  | 3’ | 13 | 186 | [152-(4)-30] |
| USN27034 | 13; >150 | 5’ | 13 | 180 | ND |
|  |  | 3’ | 13 | 126 | [83-(1)-7-(1)-8-(1)-8-(1)-7-(2)-7] |
| USN29249 | 5; >150 | 5’ | 5 | 184 | ND |
|  |  | 3’ | 5 | 170 | [94-(2)-7-(1)-8-(1)-6-(3)-8-(1)-6-(11)-8-(2)-13] |
| USN32388 | 14; >150 | 5’ | 14 | 204 | [133-(46)-26] |
|  |  | 3’ | 14 | 194 | [133-(1)-8-(35)-17] |

* ND: No detection of interruptions

^†^  The number of repeats was based on the presence of peaks (the approximation approach) and for large expanded alleles was the actual count until peaks diminished (without applying the cut-off) to facilitate the analysis of non-CTG repeats as mentioned in Materials and Methods.

^‡^ This sample was additionally tested using Sanger sequencing to contain (CTG)_6_(CCGCTG)_14_(CTG)_5_.
